# A cycle of brain gain, waste and drain - a qualitative study of non-EU migrant doctors in Ireland

**DOI:** 10.1186/1478-4491-11-63

**Published:** 2013-12-09

**Authors:** Niamh Humphries, Ella Tyrrell, Sara McAleese, Posy Bidwell, Steve Thomas, Charles Normand, Ruairi Brugha

**Affiliations:** 1 Department of Epidemiology and Public Health Medicine, Royal College of Surgeons in Ireland, Dublin, Ireland; 2 Centre for Health Policy and Management, Trinity College Dublin, Dublin, Ireland

**Keywords:** Doctor migration, Medical workforce planning, Health workforce planning, Human resources for health

## Abstract

**Background:**

Ireland is heavily reliant on non-EU migrant health workers to staff its health system. Shortages of locally trained health workers and policies which facilitate health worker migration have contributed to this trend. This paper provides insight into the experiences of non-EU migrant doctors in the Irish health workforce.

**Method:**

In-depth interviews were conducted with 37 non-EU migrant doctors in Ireland in 2011/2012.

**Results:**

Respondents believed they had been recruited to fill junior hospital doctor ‘service’ posts. These posts are unpopular with locally trained doctors due to the limited career progression they provide. Respondents felt that their hopes for career progression and postgraduate training in Ireland had gone unrealised and that they were becoming de-skilled. As a result, most respondents were actively considering onward migration from Ireland.

**Discussion & conclusions:**

Failure to align the expectations of non-EU migrant doctors with the requirements of the health system has resulted in considerable frustration and a cycle of brain gain, waste and drain. The underlying reasons for high mobility into and out of the Irish medical workforce must be addressed if this cycle is to be broken. The heavy reliance on non-EU migrant doctors to staff the medical workforce has distracted from the underlying workforce challenges facing the Irish medical workforce.

## Background

### Reliance on non-EU migrant doctors

In recent years, Ireland has developed a reliance on migrant health workers to staff its health system [1-5]. In this regard, Ireland is similar to several other high income countries, categorised by Kuhlmann et al., as ‘Anglo-American countries’ [6], which share a heavy reliance on migrant health workers [6]. Ireland merits inclusion in this category, as it had the second highest reliance on migrant doctors and the highest reliance on migrant nurses in the Organisation for Economic Cooperation and Development (OECD)^a^ in 2008 [7]. Other OECD countries with a heavy reliance on migrant health workers include New Zealand, the UK, the USA and Australia [7].

The proportion of doctors on the Irish Register of Medical Practitioners (the Medical Register) who trained in a non-EU country increased from 7.4% (N = 972) in 2000 to 25.3% (N = 4740) in 2010 [5], indicating a steep upward trajectory in Ireland’s reliance on non-EU migrant doctors. As of 2012, 23% of doctors on the Medical Register were non-EU trained, and a further 4% were non-EU citizens trained in Irish medical schools (Medical Council Ireland unpublished) (see Table 1 for definitions). In 2010, public sector vacancies for junior hospital doctors prompted an active international recruitment campaign to India and Pakistan by Ireland’s national Health Service Executive (HSE) [8], resulting in the recruitment of 290 doctors [9]. Similar recruitment campaigns targeting Pakistan and South Africa were conducted again in 2013 [10]. Active international recruitment campaigns account for a relatively small proportion of Ireland’s non-EU migrant doctors, most of whom migrated to Ireland independently.

**Table 1:** Terminology

	
**Consultant**	Doctors who have completed all of their undergraduate and postgraduate training (initial and higher specialist training) and have obtained a CSCST (certificate of satisfactory completion of specialist training) which enables them to register on the Specialist Register.
**Intern**	Doctors who have completed their basic medical degree and must complete 12 months training in an approved public hospital.
**Irish Medical Organisation (IMO)**	The national professional organisation representing medical doctors
**Non Consultant Hospital Doctor (NCHD) or Junior Hospital Doctor**	Doctors who have completed their basic medical degree and their intern year and now work in a hospital. Some NCHDs are also undertaking postgraduate training (initial or higher specialist training). Those who work as NCHDs but are not involved in formal postgraduate training are said to occupy ‘service posts’. Senior House Officers (SHOs), Registrars and Specialist Registrars are considered NCHDs or junior hospital doctors.
**Non-EU migrant doctor**	For the purpose of this study, the term non-EU migrant doctor refers to those who trained in non-EU countries and also those doctors from non-EU countries who trained in Ireland and remained to work in the Irish health system.
**Specialist Registrars (SpRs)**	Doctors who have completed their basic medical degree, their intern year and their postgraduate training (initial and higher specialist training). Specialist Registrars are working towards their CSCST (certificate of satisfactory completion of specialist training) in order to compete for a consultant post.

Two factors facilitated this migration flow in the Irish context – the insufficient availability of locally trained health workers and new immigration policies designed to facilitate the inward migration of health workers. As Kapur and McHale explain, ‘the barriers facing the highly skilled are coming down as richer countries see economic and demographic advantage in buttressing their talent and taxpayer ranks’ [11]. Specific migration routes for skilled migrants have recently been opened in France, Germany and the UK, joining countries such as Canada, Australia and the USA as countries encouraging high skill migration [12]. Significant changes to migration policy occurred in Ireland from 2000 onwards which have facilitated the migration of migrant health workers to Ireland [1,5]. These changes have been seismic in nature and, since 2012, doctors from outside the EU coming to fill specific posts in the Irish public health system no longer require working visas [5,13]. This policy change was unprecedented in the Irish context and indicates the importance of doctor migration to the Irish health system.

### Training and retaining doctors

With regard to the second factor – the insufficient availability of locally trained health workers, Ireland, along with the USA [14] and Australia [15], has traditionally trained too few doctors to meet demand and relied upon international recruitment, to meet the shortfall. From 1978 to 2007/2008, Ireland ‘capped’ the number of medical school places available to Irish/EU students at 305 [16]. This number is far below the 700 to 740 Irish/EU medical graduates per annum required to achieve self-sufficiency [16]. Although additional medical school places were available in Irish medical schools during that time, these were reserved for non-EU students who paid higher fees than EU students [16].

Beginning in 2007, Graduate Entry Programmes (GEP) were established in Ireland [17] with the express purpose of increasing the number of Irish/EU medical graduates. The number of medical graduates has increased as a result - from the baseline of 305 Irish/EU medical graduates to 448 in 2011 and 640 in 2012 (HEA unpublished statistics). In 2010/2011, there were 750 Irish/EU entrants to medical school (both undergraduate and postgraduate) (HEA unpublished statistics), indicating that the number of locally trained Irish/EU doctors will meet the levels required to achieve national self sufficiency in the coming years. This should reduce Ireland’s reliance on non-EU migrant doctors to staff its medical workforce.

In the medium term, the increased number of doctors trained in Ireland has not eased the recruitment challenges facing the health system, indicating an underlying problem of retention. This correlates with Buchan and Aiken’s assertion that a shortage may not indicate a shortage of suitably skilled and qualified people, but rather the unwillingness of those skilled individuals to work under the available conditions [18]. As Ireland now trains sufficient doctors locally to staff its medical workforce, the continuing struggle to fill junior hospital doctor posts indicates a deeper malaise. Recent moves by Ireland’s junior hospital doctors to initiate industrial action [19] is another indication of dissatisfaction within the wider medical workforce.

### Non-EU migrant doctors within the Irish health system

**Figure 1:**
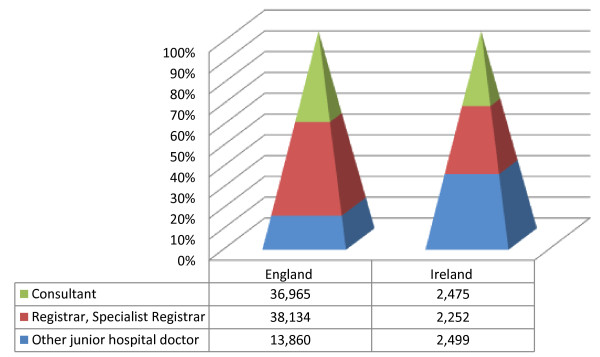
**Medical posts in England and Ireland, 2011 **[24]**,**[25]**.**

In Canada, migrant doctors occupy hard-to-fill posts, particularly those in remote locations [20]. The same is true internationally [21]. In the Irish context, it has been said that non-EU migrant doctors are hired simply ‘to wear white coats and fill gaps*’*[22]. The hard to fill posts in the Irish context are the ‘service posts’ which are not connected to formal postgraduate training schemes. As can be seen from Figure 1, the Irish health system relies more heavily on junior hospital doctors than the English health system. There are more junior hospital doctors in Ireland than there are Consultant posts for them to work towards, meaning that many posts do not offer a pathway to achieving consultancy. Doctors occupying these unpopular ‘service posts’ have limited opportunities for career progression and as of 2011, accounted for 1278 of the 4751 junior hospital doctor posts in the public health system [8]. The latest available data (from 2007) indicate that non-Irish doctors are more likely than Irish doctors to occupy these ‘service posts’ [23].

Drawing on in-depth qualitative interviews with 37 non-EU migrant doctors, this paper seeks to deepen our understanding of their expectations and experiences of the Irish health system. As such, these insights will be an invaluable aide to health workforce planners seeking to understand the movement of non-EU migrant doctors into and out of the medical workforce. Some of the issues faced by respondent non-EU doctors are system-wide issues, which also affect Irish and EU doctors in the system, while other issues are more specific to the non-EU migrant doctor workforce. As newcomers, migrants frequently test the comprehensiveness of existing policies, procedures and processes of the destination country [26]. Respondent non-EU migrant doctors provide insights into the human consequences of policy choices [27] and may well elicit similar experiences from other countries that are major recruiters of migrant doctors. Their experiences may also provide more general insight into the role of the junior hospital doctor within the Irish health system.

## Methods

### Data collection

Ethical approval for the study was received from Trinity College Dublin in 2011. In-depth interviews were conducted with 37 non-EU migrant doctors in late 2011/early 2012. A total of 35 of the interviews were conducted in person and 2 via the telephone. Respondents were recruited in three main ways: via the Irish Medical Directory [28], through an advertisement in the Irish Medical Times and via a non-governmental organisation working with immigrants in Ireland. Respondents who had taken part in a previous academic study on non-EU migrant doctors in Ireland were also invited to take part. Snowball sampling, a process of chain referral whereby respondents and gatekeepers are used to refer the researcher to other potential respondents [2,29], was also used. The researchers sought to include doctors from a range of non-EU countries and, as much as possible, to have the sample reflect the profile of internationally trained doctors registered to practice in Ireland and this objective was largely achieved.

Most respondents (25/37) listed Pakistan, Sudan, India or Nigeria as the country in which they received their primary medical training in proportions comparable with the wider population of internationally trained doctors registered in Ireland [30]. According to the World Health Report 2006, three of these countries are experiencing critical shortages of health workers and the fourth, Sudan, has the lowest physician ratio with only 0.22 physicians per 1000 population [31].

When recruiting respondents, researchers also sought to include respondents who varied according to their year of arrival, grade level and work location in Ireland. A total of 10 respondents were Dublin-based at the time of the interview, 16 respondents were based in locations nationwide and one respondent had recently emigrated from Ireland. A limitation of the sample is that it under-represents doctors trained in South Africa and over-represents doctors from Australia and the United States [30].

### Respondent profile

Respondents were all non-EU migrant doctors who were either working (n = 33), had recently worked (n = 2) or who were seeking work (n = 2) in the Irish health system. Most had undertaken their basic medical training in a non-EU country, although two had undertaken basic medical training in Ireland. A total of eight were Irish citizens, having become naturalised since arrival, while others held dual nationalities. Of the respondents, 13 were female and 24 were male. Respondents ranged in age from 29 to 70 years with an average age of 41. In terms of their grades within the Irish health system, 7 were consultants, 24 were junior hospital doctors and 2 were GPs. Respondents had graduated from medical school on average 5.7 years prior to their arrival in Ireland and 31/37 respondents had arrived in Ireland since 2000.

### Theme sheet development

The development of the interview theme sheet was informed by a process of consultation with the international literature on doctor migration, consideration of the themes discussed with non-EU migrant nurses in a previous project on nurse migration involving two of the authors [2,3] and a series of preparatory meetings with key stakeholders to obtain insights into the experiences of non-EU migrant doctors in Ireland. The themes were also central to the aim of the Doctor Migration Project, which was to explore the experiences of non-EU migrant doctors living and working in Ireland, ascertain their future plans and assess the implications for workforce planning in Ireland.

### Interviews and data analysis

Interviews explored respondents’ experience of migration in a number of stages, from pre-migration through to future intentions. Respondents were asked about the factors influencing the decision to migrate to Ireland, their expectations on arrival to Ireland, and their experiences of practicing medicine in Ireland, with a particular emphasis on postgraduate training and career progression. Respondent’s future plans were explored in terms of their intent to remain in Ireland or emigrate elsewhere and the factors influencing those decisions. Interviews were audio recorded and transcribed in full. Interview transcripts were coded and re-coded several times by two researchers, moving from thematic coding to analytic coding [32]. Management of the coding process was supported by MaxQDA software for the analysis of qualitative data.

### Limitations

A challenge for the research team was that, at the time of interview, minimal profile information was available on the non-EU migrant doctor workforce in Ireland, beyond their country of training [33]. This made it difficult to recruit a respondent grouping that was representative of the wider non-EU migrant doctor population in Ireland. Recent information published by the Medical Council [30], which includes the speciality and age profile of internationally trained doctors, will greatly assist future research in this field. A limitation of the sample is that it under-represents doctors trained in South Africa and over-represents doctors from Australia and the United States when compared with the wider population of internationally trained doctors registered in Ireland [30].

## Results

The research findings provide an insight into Ireland’s health system from the perspective of non-EU migrant doctors working in Ireland. Respondents described the inward migration of non-EU trained doctors, i.e., the arrival of highly skilled migrant doctors to Ireland. This is classified as a brain gain from source countries to the Irish health system. Respondents detailed the de-skilling process, whereby some of those non-EU doctors who have migrated to Ireland encounter limited training opportunities and stalled career progression in the Irish health system – a process classified as brain waste. Finally, respondents highlight the emigration intentions of non-EU doctors dissatisfied with their career progression – indicating a second wave of doctor emigration from Ireland and another case of brain drain.

### Brain gain: inward migration of doctors

Respondents considered that the demand for non-EU migrant doctors in Ireland was driven by the need to fill vacant, service (i.e., non-training) posts within the Irish health system.

‘They are trained there and we are getting them here just to cover the gap that we have from the system, and that’s it. . . we are just bringing them to. . . give them the hard work’ (Doctor 18).

‘Non-EU doctors . . . keep the system up and running’ (Doctor 2).

Parallels can be drawn with the experiences of non-EU migrant nurses [4], who also perceived that their recruitment was to fill gaps within the system. Although the migration of doctors to Ireland represents an injection of considerable medical skills and experience into the health workforce, respondents felt that they had been recruited to work in posts with working conditions unacceptable to Irish-trained doctors, as this respondent explains

‘they didn’t have a choice, they just need us and that’s why they have to appoint us, that’s my feeling after being here for ten years’ (Doctor 23).

Another common theme in the interviews was whether or not retention-focused measures would be a more cost effective way of managing the medical workforce than doctor migration. This arose specifically in relation to the recent active international recruitment campaigns [5,34]. Respondents felt that retaining doctors already working in the Irish health system (Irish, EU and non-EU doctors) could be a more cost-effective solution for the Irish health system.

‘The cost of people going over there, interviewing everybody, bring the doctors here, ticket, flights, all fixed expenses. . . if you could improve the conditions of the doctors you are having you wouldn’t have gotten into this problem at the first place’ (Doctor 24).

‘this year India and Pakistan and brought a few doctors from there, but what about those who are already here, why don’t you keep them here?’ (Doctor 19).

### Brain waste

As outlined earlier, a feature of the Irish health system is its reliance on junior hospital doctors (see Figure 1). Vacancies tend to be at the junior hospital doctor level, particularly ‘service posts’ which are not linked to formal postgraduate training schemes. These posts, which account for one in five junior hospital doctor posts [35], contain a disproportionate number of non-EU migrant doctors [36] and offer limited opportunities for career progression.

Salt defines brain waste as the de-skilling that occurs when highly skilled workers migrate into forms of employment that do not require the levels of skills and experience they had applied in their former post [37]. Experiences of brain waste and stalled career progression ( ( Humphries N et al. ‘I am kind of in stalemate’. The experiences of non-EU migrant doctors in Ireland. In Buchan J, Wismar M, Glinos I, Bremner, J eds. *Health professional mobility in a changing Europe: new dynamics, mobile individuals and policy responses*. European Observatory Studies Series. WHO Forthcoming 2014). were reported by respondents who felt that their medical careers were not progressing in Ireland, that they were becoming de-skilled. This was particularly true for those working in service posts which exist outside formal postgraduate training and career progression structures and which offer little or no opportunity for up-skilling. Respondents described their experiences in these posts as ‘filling in the blanks’ (Doctor 5), ‘not moving forward’ (Doctor 27) and of being a ‘labourer’ (Doctor 16).

*‘*I know that we are doing a service job, it is just 100% a service job’ (Doctor 26).

Respondents who occupied these roles felt that they were becoming de-skilled in Ireland.

*‘*When I came here I thought that when I go back home I should be able to do something more . . . I should carry some skills back home. But unfortunately if you see the graph it has started decreasing down, and it is coming down’ (Doctor 3).

‘Yes I had a great expectations, I thought . . . they will train me. Rather than training me I have been losing my skills you know, so it was a really, really upsetting’ (Doctor 3).

The frustration and hopelessness felt by these by these non-EU migrant doctors with regard to their professional development and career progression was palpable, a feeling that; ‘we don't have any future . . . don't have any hope’ (Doctor 16). Several respondents mentioned that they would not recommend Ireland as a suitable location for non-EU migrant doctors, largely because of the risk of becoming de-skilled.

‘Not to come to Ireland. . . This is not just my advice; this is the advice from everybody here, even from people in this country. If you want to go and make a good career out of it, good training, you must go to a different country, not to Ireland; it is just a waste of time here’ (Doctor 21).

Junior hospital doctor posts, both training and service posts, also require frequent moves between hospitals every 6 or 12 months, a process known as ‘rotation’. Non-EU migrant doctors working in service posts felt trapped, both in terms of the rotation system and in terms of their career progression.

‘They could be working as Registrars for 10 years in Ireland and will never get a Consultant post and I think that is not fair . . . They have families, they have to move every 6 months or every year, it is just not right’ (Doctor 18).

Respondents felt that the Irish health system should be up front with its non-EU migrant doctors and potential migrant doctors about the opportunities (or lack of opportunities) available to them in Ireland. Although there are employment opportunities in Ireland, there are limited opportunities for training or career progression and, because of the rotation system, involve frequent moves. There was a feeling that there was a level of misinformation in the recruitment process, as this respondent explains.

‘since they are lacking the doctors, to attract the doctors they are . . . manipulating the things. They are not giving true picture, they are trying to hide the things, they are giving the good picture and then not showing them the dark side’ (Doctor 7).

For those non-EU migrant doctors who were actively recruited to Ireland in 2011, assurances that were reportedly given to them regarding the continuation of their postgraduate training in Ireland were unrealised. As a result, these recruits found their career progression in Ireland stalled [38,39]. The discrepancy between the ambitions of non-EU migrant doctors and the service needs of the Irish health system meant that dissatisfaction was inevitable for many. ( Humphries N et al. ‘I am kind of in stalemate’. The experiences of non-EU migrant doctors in Ireland. In Buchan J, Wismar M, Glinos I, Bremner, J eds. *Health professional mobility in a changing Europe: new dynamics, mobile individuals and policy responses*. European Observatory Studies Series X WHO Forthcoming 2014)

Many respondents (26/37) were actively considering their future options in terms of having plans to migrate to another country.

### Brain drain: migrant doctors intent to emigrate

Of those 26 migrant doctor respondents who expressed intent to emigrate, the most frequently cited reasons related to career progression and the availability of postgraduate training opportunities. Dissatisfaction with the posts available to non-EU migrant doctors and the limited opportunities they presented was a recurring theme of interviews.

‘They need to make more positions for training. Or people will leave this country; because why should I waste my time here when I know that I could get a good chance in Australia or UK’ (Doctor 21).

‘I don’t think I have any career prospects, that is why we are planning to move. . . I can work of course indefinitely, but just the conditions doesn’t suit me anymore’ (Doctor 33).

‘Either you go back home at your Registrar level or you stay in Ireland and whole of your life you are a Registrar which is bad, you are not climbing up your career ladder’. (Doctor 27).

Respondents felt that they needed to move from Ireland in order to obtain the training necessary to progress their careers, citing countries such as the UK and Australia as offering better training and career progression opportunities.

‘So I think I have gained what I have gained, either I have to sacrifice and go to UK for my training to become a consultant or stay here all my life which is not good’ (Doctor 27).

The intention or decision to leave Ireland was almost always taken for reasons relating to their profession and the conditions attached to their work as a doctor in Ireland (e.g., rotation, career progression, postgraduate training). Having spent several years in Ireland already, 22/37 respondents were frustrated to find limited opportunities for career progression available to non-EU migrant doctors.

‘Good people who want to work hard, who can work, who have good skills and want to use them. In the end Irish system put a big wall in front of them and they leave Ireland’ (Doctor 4).

Even those who had managed to access postgraduate training in Ireland were convinced that they would need to leave Ireland to progress their careers and achieve a consultant post. This relates to the lack of consultant posts in the Irish health system. Although the lack of consultant posts affects all junior hospital doctors, respondents also felt that they were less likely than an Irish trained doctor to achieve career progression within the Irish health system, as these respondents explain:

‘It is just the system, you know this system itself, understand that most of us NCHDs we don't have any future’ (Doctor 16).

‘after having the SPR [Specialist Registrar] in here there are good chance in the Canada to get the consultant posts’ (Doctor 7).

‘the next level for me is consultant. If I don’t get it obviously I will have to go abroad, the place where I will get the post’ (Doctor 30).

## Discussion

The implications of these findings pose a significant challenge to the Irish health system, which in some cases actively recruited non-EU migrant doctors into the Irish health system. Having gone to significant lengths to attract non-EU migrant doctors with sufficient skills and experience into the health system and employ them, the health system fails to make optimal use of their talents and skills (brain waste). As a result, many see no alternative but to follow their Irish trained colleagues in exiting the Irish health system in order to achieve career progression.

### A system-wide problem

These accounts narrated by respondent non-EU migrant doctors paint a bleak picture of the experiences of non-EU migrant doctors within the Irish health system. In a sense, many of the issues presented in this paper are not specific to migrant doctors, but are system-wide issues also affecting Irish trained doctors which have not been resolved - ‘the conditions under which non-consultant hospital doctors work in Ireland have been an unacknowledged national emergency for years’ [40].

These conditions, along with Ireland’s failure to implement the European Working Directive^b^, led to junior hospital doctors initiating industrial action in October 2013 [19]. Both Irish trained and non-EU doctors seem to react in similar ways to the challenges of working in the Irish health system, experiencing low morale and dissatisfaction [41], which translates for some into intent to emigrate and to emigration.

The experiences of respondent non-EU migrant doctors are reflected in recent research on the wider medical workforce. A recent survey by the HSE Medical Education and Training Division of the 2010/2011 intern cohort (N = 226) reported that the emigration decisions of newly qualified doctors were influenced by lifestyle choices, dissatisfaction with medical training structures in Ireland and the greater availability of training opportunities abroad [35]. These factors were also highlighted in a survey of junior hospital doctors who cited unattractive working conditions, long working hours and poor career pathways for junior hospital doctors [42] as factors prompting the emigration of Irish trained doctors.

Comparing the experiences of non-EU migrant doctors with the experience of the wider medical workforce should not detract from the experiences of non-EU migrant doctors; rather, it highlights the fact that similar challenges are faced across the medical workforce. Therefore, solutions will be required to address some of the long-standing and intractable workforce challenges. Zander et al. found that internationally trained nurses working in Germany cited similar problems with regard to their work environments, as did German trained nurses who had emigrated from Germany [43]. Such findings should enable a broad-based response to improve working conditions and improve retention rates among migrant and non-migrant doctors.

In terms of short term solutions to some of these problems, perhaps the Irish health system should make a clearer attempt to align expectations of non-EU migrant doctors with reality by clarifying the limitations of service posts prior to recruiting non-EU migrant doctors into those posts. Perhaps the rotation system, which forces doctors to relocate every 6 to 12 months, could be modified or removed in order to provide doctors with greater stability in Ireland. Although these suggestions will not reduce the level of de-skilling or brain waste experienced by non-EU migrant doctors, as a first step towards reform, more honesty with potential new recruits may reduce the level of frustration and dissatisfaction experienced by respondents whose expectations of the Irish health system were not met.

### A cycle of brain gain, waste and drain

**Figure 2:**
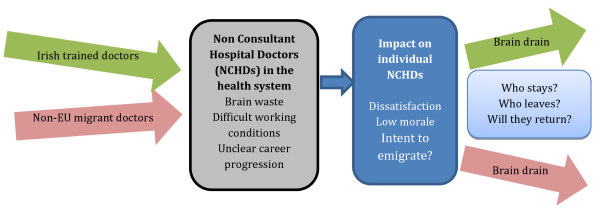
The brain gain-drain cycle in the Irish context.

**Figure 3:**
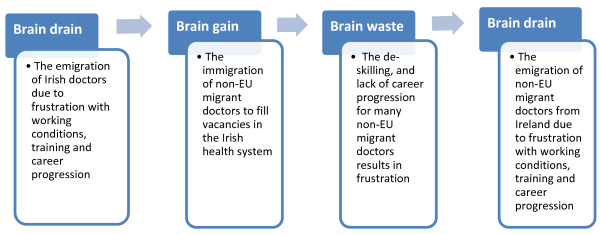
The brain gain-drain cycle of doctor migration into and out of Ireland.

Putting the experiences of non-EU migrant doctors into a wider health system context reveals a cycle of brain gain, waste and drain [20] within which the consistent losers are the individual junior hospital doctors and the Irish health system itself. The doctors struggle with difficult working conditions and uncertain career progression and the health system experiences high turnover of trained and qualified staff. The Irish Medical Organisation (IMO)^c^ has highlighted this as ‘a retention rather than a recruitment issue’ [42], as Figures 2 and 3 demonstrate. Although recruiting and training an increasing number of medical graduates will supply new doctors into the health system, a failure to improve retention will inevitably create further demand for fresh recruitment.

Figures 2 and 3 illustrate the wider health system context within which respondent experiences might be understood. Entering the health system as junior hospital doctors, the non-EU migrant doctor and the newly qualified Irish trained doctor experience many of the same challenges – long working hours, the rotation system and difficult working conditions. The impact on the individual doctor is similar, resulting in dissatisfaction and low morale, themes that emerge strongly in this paper. For many doctors, these feelings translate into intent to emigrate and emigration.

Figure 3 illustrates the chronology of the brain drain cycle in the Irish context, with two distinct ‘waves’ of doctor emigration – of Irish trained and of non-EU trained doctors. In reality, both brain gain and brain drain are daily facts of life in the Irish health system. A recent report by the Medical Council of Ireland indicates that the exit rate for non-EU migrant doctors (at 15.3% for African trained doctors and 10.4% for doctors trained in South East Asia) is higher than the average workforce exit rate of 8% [30]. The failure to retain doctors employed in the health system can be viewed as a brain drain, as it involves the loss of doctors who are trained, experienced and familiar with the Irish health system. From a systems’ perspective, it should raise alarm bells in respect to any assumptions that the inward migration of non-EU migrant doctors is a long-term solution to medical workforce shortages.

### The specific case of non-EU migrant doctors

Despite the parallels that can be drawn between the experiences of Irish and non-EU trained doctors within the Irish health system, their experiences cannot be considered identical. Respondent experience suggests that, as in other countries, de-skilling and stalled career progression is a specific feature of doctor migration – specialists can find themselves reduced to novice doctors [44], migrant doctors struggle to obtain fellowships and have limited long-term career options [45].

A major factor in the dissatisfaction of respondent migrant doctors is the feeling that they are in Ireland to fill the gaps vacated by locally trained health workers [22], although they had come to Ireland with hopes of achieving postgraduate training and career progression. There has been a failure to align the expectations of non-EU migrant doctors with the requirements of the health system. The implications of this failure are considerable, particularly for the non-EU migrant doctors themselves who have found their careers stalled in Ireland. The Irish health system, in failing to meet their hopes and expectations , contributes to the disappointment and disillusionment that leads many to consider onward migration. (A total of 26/37 of respondents expressed intent to emigrate). Another method of aligning the expectations of potential migrant doctors with the posts available in the Irish health system would be to create a permanent junior hospital doctor grade, such as the staff or specialist doctor grade [46] in the UK. Although it would not resolve the problem of brain waste, it would enable junior hospital doctors to achieve permanent status within the Irish health system while working in service posts. Perhaps this would be sufficient incentive to encourage retention and discourage onward migration.

## Conclusions

As Glinos et al. warn, ‘international recruitment . . . does not resolve the underlying causes of workforce problems’ [47] and the Irish situation provides an excellent example of this. Although effective at filling vacancies within the Irish health system, doctor migration has not resolved the problems of the health system. Instead, as this paper has demonstrated, non-EU migrant doctors frequently encounter similar problems within the Irish health system, similar to their emigrating Irish trained colleagues, allowing a cycle of brain gain, waste and drain to evolve (as depicted in Figures 2 and 3).

Skeldon [12] highlighted the fact that migration tends to be blamed for workforce planning problems that reflect a wider failure of policy [12]. Kapur and McHale discuss underinvestment by government in the required skills as a factor underpinning the need for skilled migration and perpetual crisis recruitment from overseas [11]. Ireland has used doctor migration to fill vacancies within the health system, in place of health systems and medical workforce reforms, in relation to the working conditions of junior hospital doctors and the pathways and prospects for them to achieve consultant posts [19,41]. The emphasis has been on replacing health workers, rather than changing the conditions with which they are dissatisfied. Although this has provided a short term solution, it has also fuelled the brain drain cycle outlined in Figures 2 and 3. Glinos et al. [47] emphasise retention, increased domestic supply and optimising skills and their use [47] as sustainable options for strengthening the health workforce. The attempt to rely on health worker migration to solve workforce planning weaknesses, instead of addressing retention and promoting self-sufficiency [47], is neither new nor unique to Ireland [48,49].

Ireland’s dependence on non-EU migrant doctors is neither ethical nor sustainable and is at odds with Ireland’s obligations under the WHO Global Code of Practice on the International Recruitment of Health Personnel [50]. A commitment to self-sufficiency means that Ireland should train *and* retain sufficient health workers to meet demand. This means that the high mobility characterising the Irish medical workforce (high rates of immigration and emigration) and the reasons for that mobility need to be addressed. This paper demonstrates the frustration of non-EU migrant doctors who encounter stalled career progression within the Irish health system. At some point the cycle of brain gain, waste and drain must be broken so that the high mobility currently characteristic of the Irish medical workforce is replaced with greater workforce stability.

International recruitment is an inherently short-term and unsustainable method of tackling workforce shortages. It can also distract from the underlying causes of workforce problems [47]. Once vacancies are filled, there is little impetus to analyse underlying workforce weaknesses that might have caused the vacancies [4]. Active recruitment campaigns and a steady flow of non-EU doctor migration (outside of active recruitment campaigns) have ensured that vacancies for junior hospital doctors continue to be filled, while the underlying reasons for those vacancies have not been addressed. The focus needs to move away from policies facilitating inward migration towards policies which improve the working conditions, training opportunities and career progression of junior hospital doctors and which encourage their retention in the Irish health system.

## Endnotes

^a^ Referred to in the OECD report as internationally trained doctors and nurses

^b^ The EWTD seeks to reduce the average working week to 48 hours per week

^c^ The Irish Medical Organisation is the national professional organisation representing medical doctors.

## Competing interests

The authors declare that they have no competing interests.

## Authors’ contributions

NH, RB, PB, ST and CN developed the study proposal, PB and NH carried out the data collection, NH, ET and SMcA conducted the data analysis. NH prepared all drafts and redrafts of the paper. Authors (ET, SMcA, PB, ST, CN and RB) provided editorial comment on draft versions of the paper. All authors have read and approved the final manuscript.
